# Decreased PEDF Expression Promotes Adipogenic Differentiation through the Up-Regulation of CD36

**DOI:** 10.3390/ijms19123992

**Published:** 2018-12-11

**Authors:** Kuang-Tzu Huang, Li-Wen Hsu, Kuang-Den Chen, Chao-Pin Kung, Shigeru Goto, Chao-Long Chen

**Affiliations:** 1 Institute for Translational Research in Biomedicine, Kaohsiung Chang Gung Memorial Hospital, Kaohsiung 83301, Taiwan; dennis8857@gmail.com (K.-D.C.); vina920715@gmail.com (C.-P.K.); 2 Liver Transplantation Center, Department of Surgery, Kaohsiung Chang Gung Memorial Hospital, Kaohsiung 83301, Taiwan; hsuliwen1230@gmail.com (L.-W.H.); clchen@cgmh.org.tw (C.-L.C.); 3 Fukuoka Institute of Occupational Health, Fukuoka 815-0081, Japan; pochigoto0224@gmail.com

**Keywords:** pigment epithelium-derived factor, adipogenic differentiation, adipose-derived stem cells, CD36, adipose triglyceride lipase

## Abstract

Adipogenesis is a tightly regulated cellular process that involves the action of multiple signaling pathways. Characterization of regulators that are associated with adipose development is crucial to understanding the mechanisms underlying obesity and other metabolic disorders. Pigment epithelium-derived factor (PEDF) is a secreted glycoprotein that was first described as a neurotrophic factor. The role of PEDF in lipid metabolism was established when adipose triglyceride lipase (ATGL), a major triglyceride hydrolase, was characterized as its binding partner. In this study, we investigated the downstream effects of PEDF on adipogenic differentiation using rat adipose-derived stem cells (AdSCs) and the mouse pre-adipocyte cell line 3T3-L1. Knocking down PEDF in differentiating cells resulted in elevated levels of ATGL and CD36, as well as other adipogenic markers, with a concomitant increase in adipocyte number. CD36, a scavenger receptor for a variety of ligands, regulated proliferation and lipogenic gene expression during adipogenesis. The CD36 increase due to PEDF down-regulation might be a result of elevated PPARγ. We further demonstrated that PEDF expression was regulated by dexamethasone, a synthetic glucocorticoid that is widely used for adipogenesis at the transcriptional level. Taken together, our findings highlight that PEDF negatively regulates adipogenesis through the regulation of various signaling intermediates, and it may play a crucial role in lipid metabolic disorders.

## 1. Introduction

Obesity is marked by excessive body fat accumulation. Although the adipose tissue is the primary organ crucial for energy storage in mammals, excessive fat accumulation can cause severe health issues. Obesity is one of the risk factors for various metabolic diseases such as insulin resistance, type 2 diabetes, non-alcoholic fatty liver disease, cardiovascular diseases, and even cancer [1]. The development of obesity is closely associated with increased size and triglyceride levels, as well as aberrant proliferation in adipocytes [2]. In addition, adipocytes secrete a number of protein factors known as adipokines that play critical roles in the regulation of glucose and lipid metabolism and immune responses [3,4]. Alterations in adipokine levels and chronic inflammation in the adipose tissue are key features in the development of metabolic disorders [5,6]. Therefore, understanding the mechanisms that control adipogenic differentiation and triglyceride accumulation would be an efficacious approach for developing strategies for the prevention and treatment of obesity-related metabolic diseases. 

Pigment epithelium-derived factor (PEDF) is a secreted, multi-functional glycoprotein that belongs to the serine protease inhibitor superfamily, although PEDF itself does not contain any inhibitory activity [7]. PEDF was first identified in the conditioned media of human retinal pigment epithelial cells as a neurotrophic factor, with strong neuroprotective and neurodifferentiative capacities [8]. It was later discovered that PEDF acts as a potent anti-angiogenic agent, and thereby receives much attention in cancer research [9]. The role of PEDF in lipid metabolism was established when its binding partner, adipose triglyceride lipase (ATGL), a major triglyceride hydrolase, was characterized. PEDF null mice exhibit hepatic steatosis in an ATGL-dependent manner [10]. In addition, delivery of recombinant or adenoviral PEDF attenuates adipogenic differentiation of pre-adipocytes [11,12]. Clinical studies have also demonstrated elevated PEDF levels in obese population and patients with type 2 diabetes, which are positively correlated with insulin resistance [13,14]. These associations highlight PEDF as an important metabolic regulatory protein. However, the underlying mechanism regarding insulin resistance remains incompletely understood, as studies have provided divergent results in terms of its metabolic function [15]. A number of studies have reported that PEDF elevation in metabolic syndrome may act as a countermeasure against obesity-related metabolic derangement, while others suggest otherwise [16,17,18]. Metabolic functions of PEDF remain largely unknown, and require further elucidation. 

As PEDF is highly expressed in the adipocytes, in the current study, we investigated its biological functions associated with adipogenesis. Using rat adipose-derived stem cells (AdSCs) and a mouse pre-adipocyte cell line 3T3-L1, we found that the PEDF decrease during adipogenic differentiation was accompanied by a concomitant increase in the scavenger receptor CD36, which also played a direct functional role in the adipogenic process. Our results explain from a molecular perspective that PEDF may have beneficial effects in inhibition of adipogenesis. 

## 2. Results

### 2.1. PEDF Regulates CD36 Expression during Adipogenic Differentiation

**Figure 1:**
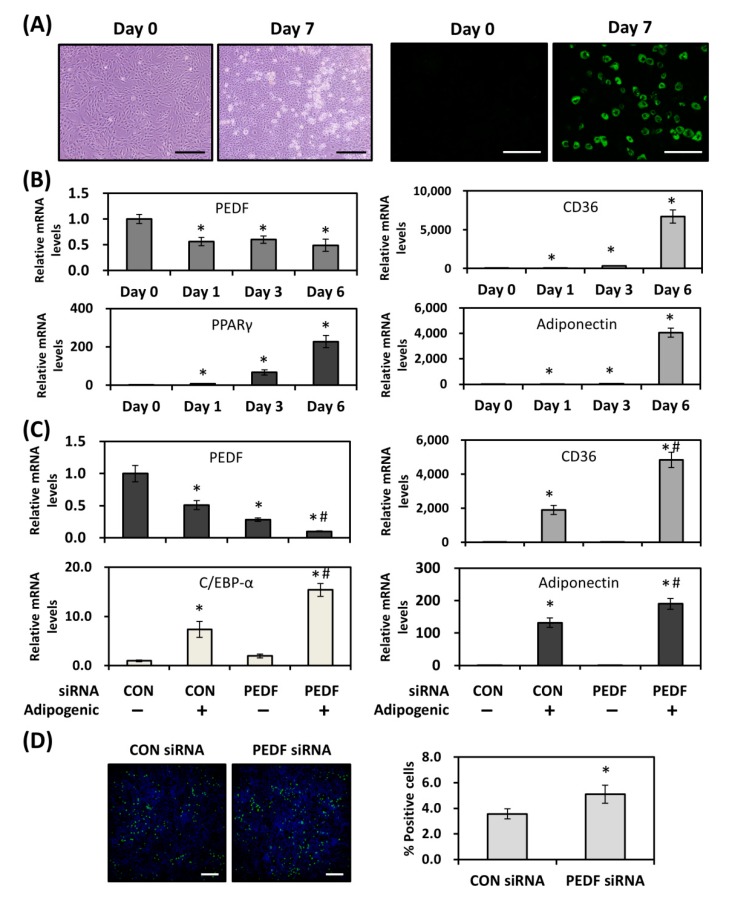
Decreased PEDF is associated with CD36 up-regulation and increased differentiated adipocyte number during the adipogenic differentiation of adipose-derived stem cells (AdSCs). (**A**) Differentiated rat AdSCs were fixed and stained with BODIPY 493/503. Representative brightfield and fluorescence micrographs of undifferentiated and differentiated AdSCs are shown. Scale bar: 100 μm (left), 50 μm (right). (**B**) Gene expression of PEDF, CD36, PPARγ, and adiponectin in differentiating AdSCs was measured using quantitative RT-PCR. (**C**) Gene expression was evaluated in differentiated AdSCs that were transfected with control or PEDF small interfering RNA (siRNA) using quantitative RT-PCR. (**D**) As in (**C**), differentiated rat AdSCs were stained with BODIPY 493/503 and counterstained with DAPI. Percent positive cells were determined by calculating the percentage of positively stained cells in all DAPI-stained cells. Scale bar: 500 μm. * Statistically significant compared with the control transfected, vehicle-treated group at *p* < 0.05; # statistically significant compared with the PEDF siRNA-transfected, vehicle-treated group at *p* < 0.05.

Up-regulation of CD36 occurs during adipogenic differentiation. The regulations between PEDF and CD36 levels during the adipogenic process were examined. Rat adipose-derived stem cells (AdSCs) were isolated, cultured, and subjected to adipogenic differentiation. A proportion of AdSCs were able to differentiate into adipocytes by day 7 (Figure 1A, left). BODIPY 493/503 staining showed evident lipid droplet formation (Figure 1A, right). Quantitative RT-PCR results revealed that expression levels of adipogenic markers were also altered accordingly (Figure 1B). PPARγ, the central mediator of adipogenesis, was gradually increased over time. Also increased was the expression of CD36 and adiponectin. Expression of PEDF, however, was decreased in response to adipogenic stimuli, suggesting a potential inhibitory role in this process. Similar observations were found by using mouse 3T3-L1 preadipocytes (representative micrograph in Figure S1A), in which PPARγ, CD36, and adiponectin were up-regulated with time, whereas PEDF was down-regulated (Figure S1B). 

With a steady decrease in PEDF expression following adipogenic stimulation, we suspected that PEDF might also have a regulatory role in adipogenesis. To demonstrate this, PEDF was knocked down in rat AdSCs and mouse 3T3-L1 cells before the induction of adipogenesis. Six days after induction in AdSCs, while a persistent decrease in PEDF and an increase in CD36 were observed, the late adipogenic gene C/EBP-α was up-regulated. Increased levels of transcription factors lead to a marked elevation of adiponectin expression. Meanwhile, the transfection of PEDF small interfering RNA (siRNA) into AdSCs decreased the mRNA levels up to 70%, and treatment with adipogenic inducers further decreased PEDF. Interestingly, knocking down PEDF resulted in a further elevation in the expression of CD36, C/EBP-α and adiponectin on day 6 of differentiation (Figure 1C). Similarly, a further increase in CD36 and adiponectin mRNA was also observed in PEDF knocked down, differentiated 3T3-L1 cells (Figure S1C). Conversely, the treatment of differentiating 3T3-L1 cells with recombinant PEDF (at 10 nM) inhibited expression of adiponectin, as well as CD36 (Figure S1D), suggesting that PEDF may directly or indirectly regulate CD36 levels and the degree of differentiation. 

To functionally demonstrate the effect of PEDF knockdown on adipogenesis, rat AdSCs transfected with control or PEDF siRNAs were assessed six days after the induction of adipogenic differentiation by BODIPY 493/503 staining. As shown in Figure 1D, the percentage of positive stained cells was increased by ~50% in PEDF knocked down AdSCs, compared with controls. 

### 2.2. Inhibition of ATGL Activity Promotes CD36 Expression and Adipogenesis

**Figure 2:**
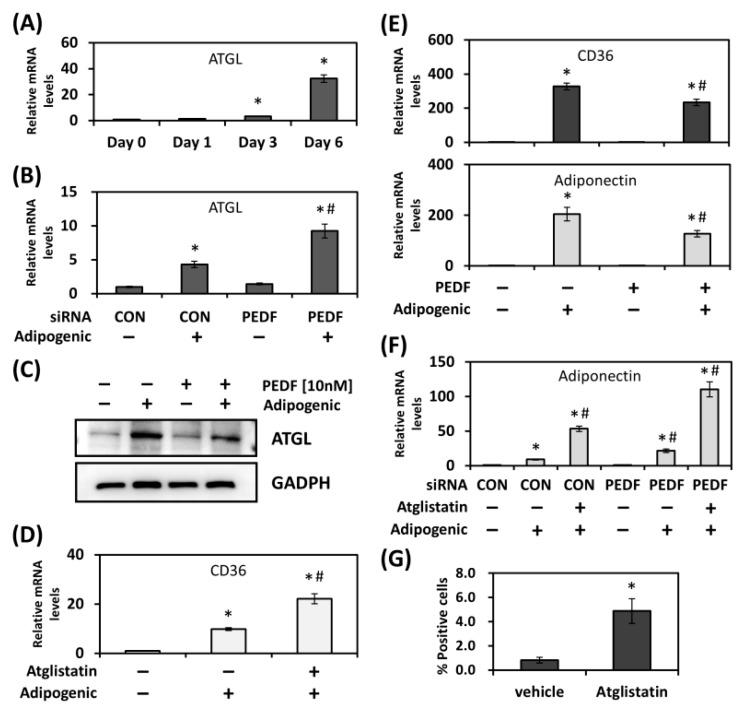
Blockade of ATGL activity promotes adipogenic differentiation. (**A**) ATGL expression was measured in differentiated rat AdSCs using quantitative RT-PCR. (**B**) ATGL expression was evaluated in differentiated rat AdSCs transfected with control or PEDF siRNA (**C**) Mouse 3T3-L1 cells induced for adipogenic differentiation were treated with recombinant PEDF. ATGL protein levels were determined using immunoblotting. (**D**) CD36 expression was measured in differentiated 3T3-L1 cells in the absence or presence of an ATGL inhibitor, Atglistatin. (**E**) Effects of PEDF on CD36 and adiponectin expression were determined in differentiated 3T3-L1 cells. (**F**) Adiponectin expression was evaluated in differentiated 3T3-L1 cells transfected with control or PEDF siRNA, in the absence or presence of Atglistatin. (**G**) As in (**D**), differentiated 3T3-L1 cells were stained with BODIPY 493/503 and counterstained with DAPI. Percent positive cells were determined by calculating the percentage of positively stained cells in all DAPI-stained cells. * Statistically significant compared with the control transfected, vehicle-treated group at *p* < 0.05; # statistically significant compared with the PEDF siRNA/PEDF protein-treated, vehicle-treated group at *p* < 0.05.

As described above, many of the PEDF-mediated functions are mainly through its binding partner, ATGL. Therefore, we next examined whether the regulation of CD36 expression and adipogenesis by PEDF was affected by ATGL enzyme activity. During adipogenic differentiation, while PEDF expression was decreased with time, we found that ATGL levels were gradually increased (Figure 2A and Figure S2A). We knocked down PEDF in differentiating rat AdSCs, and showed further increase in ATGL expression (Figure 2B). This reciprocal correlation was also corroborated with the result using immunoblotting, in which the ATGL protein level was decreased in recombinant PEDF-treated, differentiated mouse 3T3-L1 cells (Figure 2C). To show whether ATGL activity regulates CD36 expression during adipogenesis, 3T3-L1 cells were incubated with adipogenic inducers in the absence or presence of a small molecule inhibitor Atglistatin. As shown in Figure 2D, while CD36 mRNA was increased in differentiated 3T3-L1 cells, blocking ATGL activity further elevated CD36 levels. Conversely, the treatment of differentiating 3T3-L1 cells with recombinant PEDF decreased CD36 and adiponectin expression (Figure 2E). In addition to CD36, ATGL inhibitor Atglistatin also up-regulated adiponectin expression during adipogenic differentiation, which was further increased in PEDF-knocked down cells (Figure 2F). The up-regulation in gene expression can be translated into an observation that Atglistatin increased the percent positive number of differentiated 3T3-L1 cells by ~5 folds (Figure 2G). These data suggest that despite a gradual increase in ATGL expression during adipogenesis, blocking ATGL activity, in fact, has a promoting role in maintaining CD36 levels in the adipogenic process and PEDF may inhibit adipogenic differentiation via this route. 

### 2.3. PEDF Decrease during Adipogenesis Leads to Increased Levels of PPARγ, a Positive Regulator of CD36

**Figure 3:**
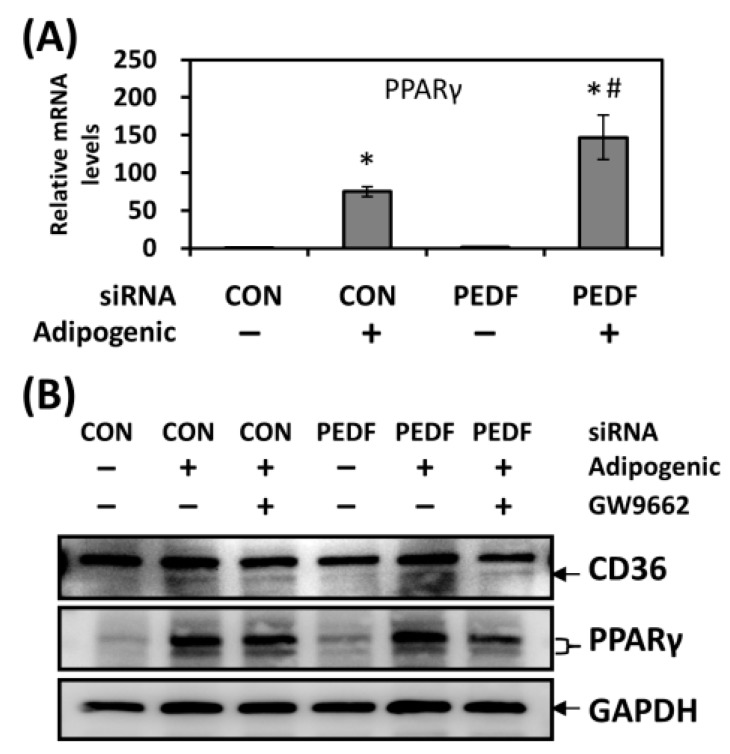
CD36 is negatively regulated by PEDF through PPARγ during adipogenic differentiation. (**A**) PPARγ expression was evaluated in differentiated rat AdSCs transfected with control or PEDF siRNA using quantitative RT-PCR. (**B**) CD36 and PPARγ protein levels were determined in differentiated mouse 3T3-L1 cells transfected with control or PEDF siRNA, in the absence or presence of a PPARγ inhibitor GW9662. * Statistically significant compared with the control transfected, vehicle-treated group at *p* < 0.05; # statistically significant compared with the PEDF siRNA transfected, vehicle-treated group at *p* < 0.05.

PPARγ, the master regulator for adipogenic differentiation, was gradually elevated, as described in Figure 1B and Figure S1B, in both rat AdSCs and mouse 3T3-L1 cells in the presence of adipogenic inducers. Knocking down PEDF in AdSCs further increased PPARγ expression in differentiating cells, suggesting that PEDF might exert its activity by modulating PPARγ levels (Figure 3A). It has been reported that CD36 is a downstream target of PPARγ [19]; we therefore examined whether the increase in CD36 upon PEDF decrease was due to elevated PPARγ activity. As shown in Figure 3B, CD36 protein was up-regulated in 3T3-L1 cells in the presence of adipogenic inducers for six days; PEDF knockdown further increased CD36 levels. When GW9662, an irreversible PPARγ inhibitor was added, CD36 was decreased in both control and PEDF siRNA-transfected conditions. These results suggest that regulation of CD36 mediated by PEDF may be through changes in PPARγ activity during adipogenic differentiation. 

### 2.4. CD36 Regulates Mitotic Clonal Expansion and Lipogenic Genes during Adipogenic Differentiation

**Figure 4:**
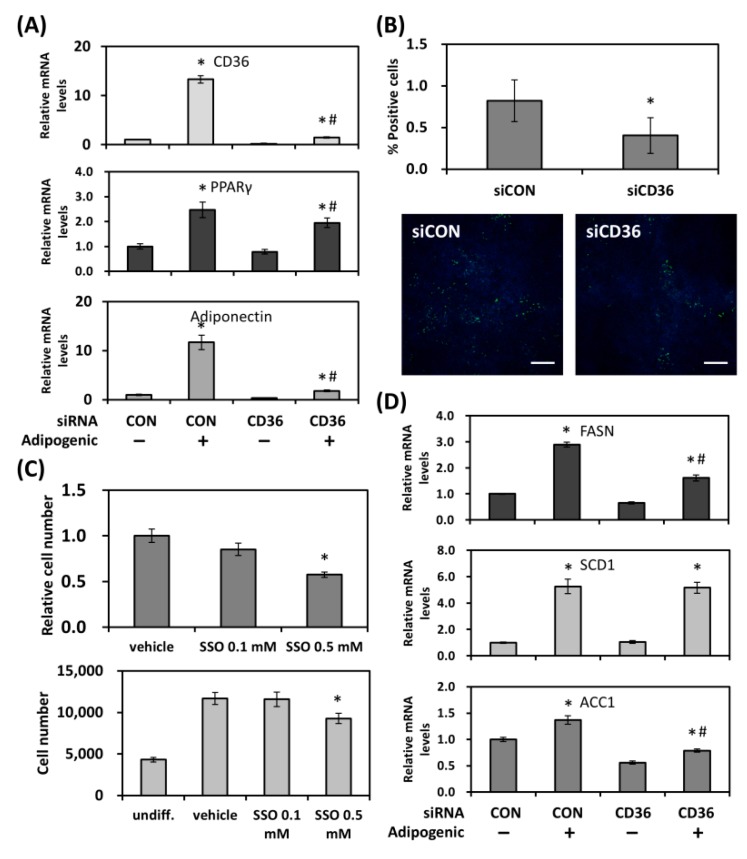
CD36 regulates adipogenesis. (**A**) Adipogenic markers were evaluated using quantitative RT-PCR in differentiated mouse 3T3-L1 cells transfected with control or CD36 siRNA. (**B**) As in (**A**), differentiated 3T3-L1 cells were stained with BODIPY 493/503 and counterstained with DAPI. Percent positive cells were determined by calculating the percentage of positively stained cells in all DAPI-stained cells. Scale bar: 500 μm. (**C**) 3T3-L1 cells were induced for adipogenic differentiation in the presence of a CD36 inhibitor SSO. Cell viability and cell number were determined after three days. (**D**) Lipogenic gene expression was evaluated in differentiated 3T3-L1 cells transfected with control or CD36 siRNA. * Statistically significant compared with the control transfected, vehicle-treated group at *p* < 0.05; # statistically significant compared with the CD36 siRNA transfected, vehicle-treated group at *p* < 0.05.

Although CD36 is a well-established marker for adipogenic differentiation, how this fatty acid trafficking protein is involved in this process remains largely unknown. To validate its involvement, CD36 was knocked down in mouse 3T3-L1 cells before the induction of adipogenesis. As shown in Figure 4A, incubation in adipogenic medium for three days resulted in a significant increase in the expression of CD36, as well as PPARγ and adiponectin. Knocking down CD36 with specific siRNAs caused a small decrease in PPARγ, but a drastic reduction in adiponectin, which reflected on the number of differentiated 3T3-L1 cells, as we observed a ~50% decrease in percent BODIPY 493/503-stained cells (Figure 4B). Potential mechanisms were also investigated. After adipogenic induction, growth-arrested 3T3-L1 cells re-enter the cell cycle and undergo two to three rounds of mitosis, the so-called mitotic clonal expansion, an essential step for terminal differentiation [20]. To examine whether CD36 was involved in mitotic clonal expansion, a specific inhibitor for CD36, sulfo-N-succinimidyl oleate (SSO), was added to the 3T3-L1 culture along with the adipogenic inducers. Three days after the induction of differentiation, cell viability and cell number were determined. The CCK-8 assay showed a ~45% decrease in cell viability in the presence of a higher dose of SSO (0.5 mM). Direct cell counting also showed a reduced cell number at the same concentration (Figure 4C). Although CD36 is best known for its role in fatty acid uptake, at the initial phase of adipocyte differentiation, the fatty acids used for triglyceride formation are mainly from de novo biosynthesis. We therefore examined whether changes in CD36 levels would affect the expression of genes involved in de novo lipogenesis and fatty acid modification pathways, including fatty acid synthase (FASN), stearoyl-CoA desaturase 1 (SCD1) and acetyl-CoA carboxylase 1 (ACC1). As shown in Figure 4D, adipogenic inducers resulted in a 3-fold increase in FASN gene expression in 3T3-L1 cells, while knocking down CD36 inhibited this increase. Similar observations were found for ACC1, as CD36 knockdown decreased its gene levels. Interestingly, CD36 silencing also decreased the basal level of FASN and ACC1 in the absence of adipogenic inducers. Nonetheless, while there was a 5-fold increase in SCD1 expression during adipogenic differentiation, knocking down CD36 was without an effect on its levels, suggesting that effects of CD36 on promoting adipogenesis are, at least in the initial phase, via the modulation of the cell cycle and specific key genes. 

### 2.5. Glucocorticoids Regulate PEDF Transcription during Adipogenesis

**Figure 5:**
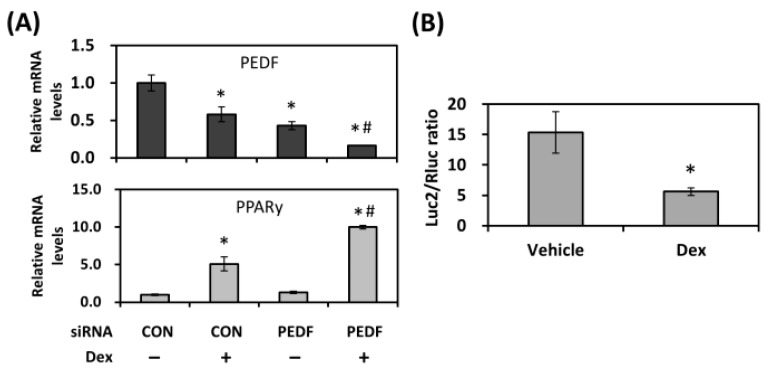
PEDF is down-regulated by dexamethasone during adipogenesis. (**A**) Rat AdSCs were transfected with control or PEDF siRNA, followed by incubation with dexamethasone (Dex) for six days. Gene expression was evaluated using quantitative RT-PCR. (**B**) Rat AdSCs were transfected with a PEDF reporter construct and treated with Dex for two days. Cells were then subjected to dual luciferase assays. Data are presented as relative mean ratios (firefly/Renilla luciferase units) ± SD. * Statistically significant from the controls at *p* < 0.05; # statistically significant compared with the PEDF siRNA transfected, vehicle-treated group at *p* < 0.05.

We showed that decreased PEDF resulted in an increase in adipogenic differentiation. Therefore, potential mediators that negatively regulated PEDF were investigated. Glucocorticoids have been implicated in the pathogenesis of non-alcoholic fatty liver disease (NAFLD) across various stages, acting on both liver and adipose tissues. Dexamethasone (Dex), a synthetic glucocorticoid agonist, together with insulin and 3-isobutyl-1-methylxanthine (IBMX), are commonly used for adipogenesis in culture. Although glucocorticoids are not strictly required for differentiation, they are capable of strongly enhancing the transcriptional activation of C/EBPs and PPARγ [21]. Using the JASPAR CORE database [22], we found two putative response elements for glucocorticoid receptor binding in the promoter region of PEDF (at −1600 and −794, respectively). Therefore, we evaluated whether glucocorticoids affected the expression of PEDF. Treatment of rat primary AdSCs with Dex alone for six days decreased PEDF expression, with a concomitant increase in PPARγ levels. Knocking down the PEDF transcripts resulted in a marked decrease in PEDF. The Addition of Dex further reduced PEDF expression and raised PPARγ levels (Figure 5A), a result that is very similar to what we observed using adipogenic inducers that also contained insulin and IBMX (Figure 1C and Figure 3A). To determine whether Dex directly affected the transcriptional activity of PEDF, we produced a luciferase reporter construct containing a 1.7 kb promoter region of the PEDF gene. The construct was transiently transfected into rat AdSCs, and relative luciferase activity was measured following treatment with Dex for 48 hr. As shown in Figure 5B, PEDF promoter activity was reduced by ~60% in the presence of Dex, indicating direct regulation at the transcriptional level. 

### 2.6. Decreased PEDF Causes an Increase in β-Catenin Phosphorylation during Adipogenesis 

**Figure 6:**
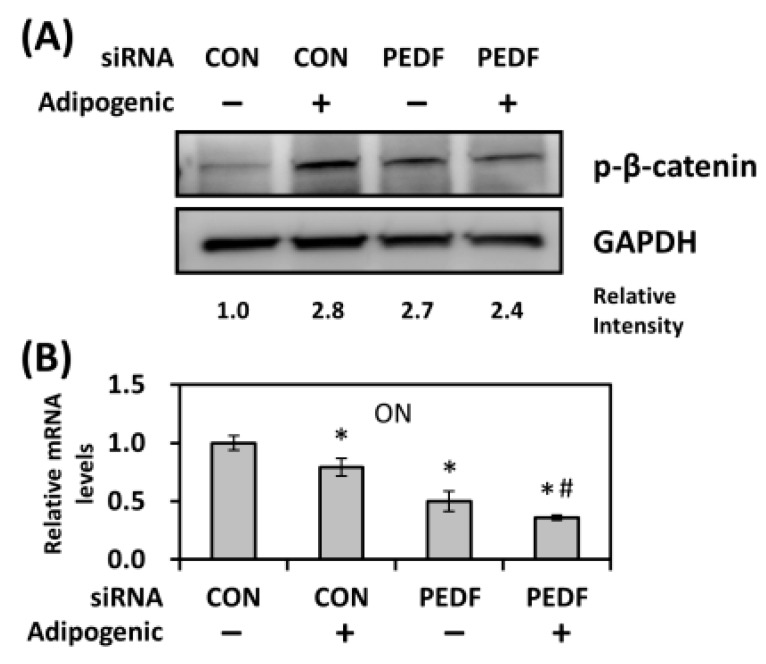
PEDF knockdown induces β-catenin phosphorylation during adipogenesis. (**A**) Rat AdSCs were transfected with control or PEDF siRNA, followed by adipogenic differentiation induction for 24 hr. Whole-cell extracts were immunoblotted for phospho-β-catenin. Relative intensity was compared with control siRNA transfected, undifferentiated cells, as determined by densitometry normalized to GAPDH. (**B**) As in (**A**), control and PEDF siRNA-transfected AdSCs were incubated with adipogenic differentiation medium for 24 hr. Gene expression was analyzed using quantitative RT-PCR. * Statistically significant from the control transfected, vehicle-treated group at *p* < 0.05; # statistically significant compared with the PEDF siRNA transfected, vehicle-treated group at *p* < 0.05.

The Wnt/β-catenin pathway has been shown to inhibit adipogenic differentiation by maintaining pre-adipocytes in an undifferentiated state, as well as to activate the osteogenic pathway [23,24]. To address whether decreased PEDF expression affected nuclear β-catenin levels during adipogenesis, AdSCs transfected with control or PEDF siRNA were incubated in adipogenic differentiation medium for 24 hr. In control transfected AdSCs, adipogenic inducers elevated the phosphorylation of β-catenin, indicating that more nuclear portions of β-catenin being degraded. Interestingly, knocking down PEDF also increased β-catenin phosphorylation, even in the absence of adipogenic inducers (Figure 6A), suggesting that AdSCs with decreased PEDF were more inclined towards adipogenesis. With the same setting, we further examined the effect of PEDF knockdown on an early osteogenic marker osteonectin (ON). Treatment with adipogenic inducers caused a ~25% decrease in ON expression; knocking down PEDF further down-regulated ON levels (Figure 6B). Late osteogenic markers such as osteopontin and osteocalcin were not detectable (not shown). 

## 3. Discussion

**Figure 7:**
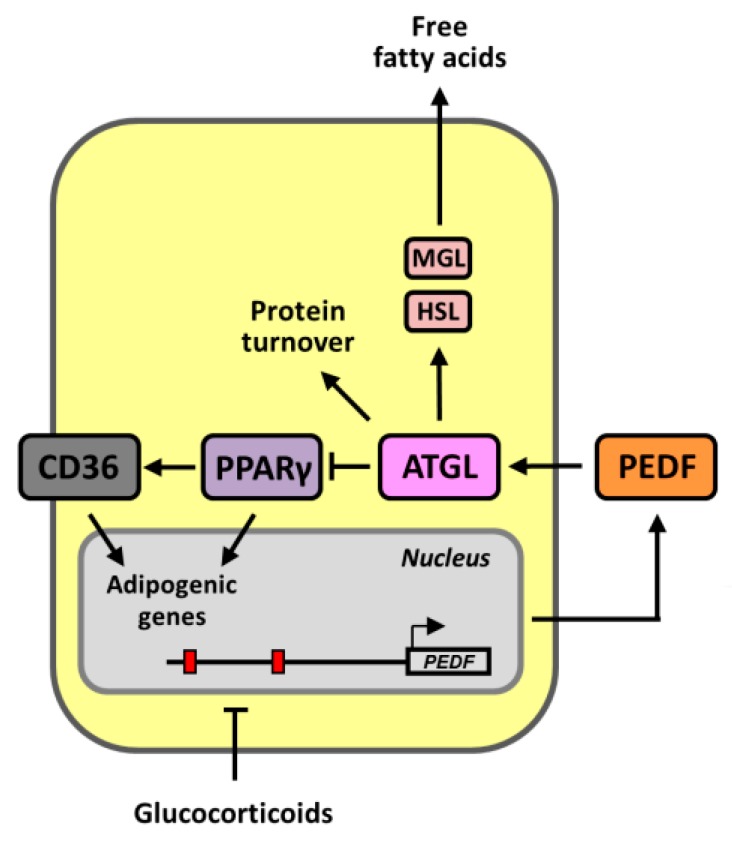
Schematic representation demonstrates the functional roles of PEDF in adipogenic differentiation. PEDF inhibits adipogenic differentiation via its binding partner ATGL (a hydrolase that breaks down triglycerides to release free fatty acids), suppressing PPARγ activity and its downstream adipogenic gene expression. PEDF also down-regulates CD36, which itself is a downstream target of PPARγ, and involved in adipogenesis. Furthermore, PEDF regulates ATGL levels by promoting its protein turnover, which may be part of a feedback mechanism. HSL, hormone-sensitive lipase; MGL; monoglyceride lipase.

PEDF, since its discovery in the retinal pigment epithelial cells as a neurotrophic factor, has been validated to be a multi-process protein in various tissues. These distinct biological effects may be derived from signaling via different binding partners and their associated intermediate proteins. The crucial role of PEDF in lipid metabolism stemmed from the studies in which PEDF null mice captured features of the metabolic syndrome in the liver and adipose tissue such as liver steatosis, increased adiposity and impaired glucose tolerance [10,17]. However, how PEDF modulates adipogenesis and intracellular fatty acid accumulation has not been well established. In the present study, we investigated the role of PEDF and its potential downstream effects in adipogenesis. Our current data showed that PEDF levels were decreased during adipogenic differentiation, with a concomitant elevation in the scavenger receptor CD36, through a PPARγ-dependent mechanism. CD36 also participates in differentiation, by modulating mitotic clonal expansion in the initial phase and regulating expression of lipid biosynthesis genes (Figure 7). 

Differentiation of pre-adipocytes to mature adipocytes is a multi-step process that is accompanied by the highly regulated induction of CCAAT-enhancer-binding protein (C/EBP) transcription factors and PPARγ, followed by activation of a variety of adipocyte-specific genes that lead to further differentiation [25]. Our data suggested a decrease in PEDF during the differentiation process. This decrease occurred as early as one day after adipogenic induction, and the expression remained at similar levels throughout. Knocking down PEDF mimicking a further decrease resulted in a more elevated expression of adipogenic markers and adipocyte numbers. Others have reported similar findings by the administration of recombinant or adenoviral PEDF to pre-adipocytes, and observed an inhibition of differentiation [11,12]. The mechanism of action has yet to be determined. However, it has been suggested that the inhibition of adipogenesis by PEDF treatment is associated with the activation of Wnt/β-catenin signaling, thereby suppressing PPARγ activity [11]. Our results also showed that by knocking down PEDF, decreased levels of nuclear β-catenin and the early osteogenic marker ON were observed. The accompanying increase in PPARγ further caused an elevation in CD36 and the subsequent stimulation of adipogenesis. 

The mechanisms by which PEDF exerts its lipid metabolic functions are thought to be through the activation of ATGL. ATGL specifically hydrolyzes the first ester linkage of triglycerides in step-wise lipolysis, followed by the action of activated hormone-sensitive lipase (HSL), releasing free fatty acids from the adipose and other lipid storage tissues [26,27]. PEDF has been shown to co-localize with ATGL at the surface of adiposomes in hepatocytes [10]. ATGL-null mice also share similar phenotypic changes with PEDF knockouts, including enlarged fat deposits and fatty liver [28]. ATGL is found at high levels in the adipose tissue. However, how ATGL is involved during adipogenesis is not well understood. Our data suggest a low expression of ATGL in undifferentiated cells and a gradual increase upon differentiation (Figure 2A). Knocking down PEDF further increased ATGL expression, whereas treatment with recombinant PEDF resulted in an opposite effect (Figure 2B,C). One potential mechanism was that PEDF treatment resulted in sustained ubiquitin-dependent ATGL protein turnover (decrease in protein stability) [29]. In addition, increased PPARγ activity following PEDF knockdown can also elevate ATGL transactivation [30]. Interestingly, despite an increase in expression during adipogenesis, our results showed that blocking the hydrolase activity by the ATGL inhibitor Atglistatin promoted differentiated cell numbers, suggesting that lipolysis may have an inhibitory role in the differentiation process. As the full hydrolase activity of ATGL also depends upon levels of its cofactors, including comparative gene identification-58 (CGI-58) and G0S2 and their subcellular localization [31], the increase during adipogenesis may not directly translate into an overall increase in hydrolase activity, and this may suggest additional signaling functions of this PEDF binding partner. 

In addition to ATGL, increased PPARγ activity following PEDF decrease also up-regulates the fatty acid transport protein CD36. CD36 is a member of the class B scavenger receptor family with the ability to bind various ligands including oxidized low-density lipoprotein (oxLDL), long-chain fatty acids, thrombospondin, oxidized phospholipids, and collagen [32]. One of the best characterized functions of CD36 is the uptake of oxLDL by macrophages to form foam cells, a key event in the initiation and progression of atherosclerosis [33]. Emerging evidence has also suggested a crucial role of CD36 in the pathogenesis of fatty liver disease and other metabolic disorders. The increased level of hepatic CD36 expression is accompanied with elevation in fatty acid uptake and triglyceride accumulation in the liver in several animal studies and in patients with NAFLD [34,35,36]. Impaired adipogenesis has also been demonstrated in CD36-deficient mice, and CD36 knocked-down pre-adipocyte cell lines [37]. Even though CD36 is one the several established markers of adipogenesis, the mechanisms by which CD36 participates in this process remain unclear. During the initial phase of adipogenic differentiation in vitro, as the adipogenic medium was not supplemented with exogenous lipids, the fatty acid moieties in triglyceride formation are mainly from products of de novo synthesis or the modification of other fatty acids [38]. We therefore suspected that CD36 may also be involved in cellular processes other than fatty acid uptake. We observed that a blockade of CD36 activity by gene knockdown or chemical inhibition affected mitotic clonal expansion and the expression of key de novo lipogenesis enzymes. In an in vivo situation, as differentiating cells are often in a microenvironment that contains free fatty acids that are released from the lipolysis of surrounding adipocytes or lipoproteins, our results therefore suggest that CD36 may promote triglyceride accumulation through both de novo lipogenesis and fatty acid uptake. 

Glucocorticoids (GCs) are essential regulators in lipid metabolism. Chronic exposure to GCs results in weight gain as well as increased visceral obesity [39]. GCs also enhance the full differentiation of adipocytes, and they are therefore commonly used to induce adipogenesis in tissue culture systems [40]. Our data indicated that Dex alone, in the absence of insulin and IBMX, was able to up-regulate adipogenic PPARγ. Moreover, using a reporter assay, we showed that Dex directly inhibited PEDF expression at the transcriptional level. Although the promoter sequence in the construct that we used in this study was from the human origin, we did predict several putative glucocorticoid response elements in the ~2 kbp rat PEDF promoter region. Whether these are true glucocorticoid receptor binding sites needs further investigation. Overall, the mechanisms through which GCs promote adipogenesis can be, at least in part, attributed to an inhibitory effect on PEDF expression, which in turn suppresses Wnt signaling, and results in increased PPARγ and downstream adipogenic genes. Interestingly, GCs can be added to a list of steroid hormones that have been validated to regulate PEDF mRNA expression in various tissues, including androgen, estrogen, progesterone, and retinoic acids [41,42,43]. As most of these steroid hormones exert distinct biological activities in the adipose tissue, additional involvement of PEDF in these processes may be anticipated. 

Taken together, our work identifies the regulatory function of PEDF in adipogenic differentiation by modulating its downstream effector CD36 through ATGL, in a PPARγ-dependent manner. We further discovered the functional roles of CD36 in adipogenesis, by modulating mitotic clonal expansion and lipogenic gene expression. We also unraveled a new mechanism by which adipogenic inducers such as GCs can regulate PEDF expression via transcriptional repression, and in turn, affects differentiation towards adipocytes. As there is a fine balance between adipogenesis and lipolysis, how PEDF affects ATGL levels and activity in these processes, and the accessory proteins that are involved will be our next target of interest. Our study has extended the current knowledge by strengthening the physiological relevance of the PEDF-ATGL-CD36 relationship in lipid metabolism, and this may lead to further studies.

## 4. Materials and Methods

### 4.1. Cell Culture and Treatments

The mouse pre-adipocyte cell line 3T3-L1 were purchased from the American Tissue Culture Collection (ATCC; Manassas, VA, USA), and cultured in Dulbecco’s modified eagle medium (DMEM) supplemented with 10% bovine calf serum (BCS), 100 μg/mL streptomycin, and 100 U/mL penicillin (ThermoFisher; Waltham, MA, USA) at a 37 °C, 5% CO_2_ atmosphere. Dexamethasone (Dex), bromoenol lactone (BEL), GW9662 (Sigma-Aldrich; St. Louis, MO, USA), sulfo-N-succinimidyl oleaste (SSO) (Santa Cruz Biotechnology; Santa Cruz, CA, USA), and PEDF (ProSpec; East Brunswick, NJ, USA) were added to the culture at the concentrations indicated.

Adipose-derived stem cells (AdSCs) were isolated from the abdomen adipose tissue of 8-week-old Lewis rats. Tissues were minced and digested in Hanks’ balanced salt solution containing 0.25 mg/mL collagenase type II (Sigma-Aldrich) for 30 min at 37 °C with agitation (50 rpm), until a smooth consistency was reached. After centrifugation, the cellular pellet was filtered through a 70 μm cell strainer (BD Biosciences; Bedford, MA, USA) and the flow-through was resuspended in DMEM containing 10% fetal bovine serum (FBS). Isolated cells were incubated until approximately 80% confluence before plating for adipogenic differentiation. Only AdSCs subcultured up to three passages were used for adipogenic differentiation. To induce adipogenic differentiation, AdSCs or 3T3-L1 cells were plated onto 6- or 12-well plates and allowed to come to approximately 80% confluence in the growth media (DMEM with 10% FBS or BCS). Cells were then incubated in adipogenic differentiation media containing 10 μg/mL insulin, 1 μM Dex, and 0.5 mM 3-isobutyl-1-methylxanthine (IBMX) (Sigma-Aldrich) for three days, followed by incubation with 10 μg/mL insulin for another three or four days. 3T3-L1 cells were induced for differentiation according to ATCC guidelines with minor modifications: 3T3-L1 cells were grown until full confluence and incubated in growth media for additional 24 hr. Cells were then cultured in adipogenic differentiation media for three days, followed by incubation with 10 μg/mL insulin for another three or four days.

In experiments that included siRNA transfection (listed in Table S1), cells were incubated in siRNA mixture using GenMute siRNA Transfection Reagent (SignaGen Laboratories; Rockville, MD, USA) (final concentrations: 20 nM), according to the manufacturer’s instructions for 6 hr before switching to the differentiation media.

### 4.2. BODIPY 493/503 Staining

Differentiated cells were stained with BODIPY 493/503 (ThermoFisher) to visualize lipid droplet accumulation according to the manufacturer’s instructions. Briefly, cells were first fixed in 4% paraformaldehyde (PFA) for 10 min. Following appropriate washing, BOPIPY 493/503 (1 mg/mL in DMSO) was applied to the fixed cells (final concentration at 0.5 μg/mL) for 30 min. The cells were then counterstained with DAPI (0.5 μg/mL) to identify the nuclei. To evaluate the percentage of differentiated adipocytes, plates were read on the ImageXpress Micro XLS system (Molecular Devices; Sunnyvale, CA, USA) under a 4× S Flour objective. Nine images were captured for each experimental condition. Acquired cell images were analyzed using the MetaXpress software (Molecular Devices). The percentage of positive stained cells were calculated.

### 4.3. Quantitative RT-PCR

Total RNA was extracted using the RNeasy Mini Kit (Qiagen; Valencia, CA, USA) according to the manufacturer’s instructions. First strand complementary DNA (cDNA) was synthesized with 1 μg total RNA using High Capacity Reverse Transcriptase (Applied Biosystems; Grand Island, NY, USA). Quantitative RT-PCR was performed using SYBR Green PCR Master Mix with specific primers (listed in Table S2) on an ABI 7500 fast PCR system (Applied Biosystems). A melting curve analysis was carried out to ensure primer specificity. The relative gene expression was calculated against GAPDH or actin as internal controls using the ∆∆Ct method.

### 4.4. Immunoblotting

Whole cell extracts were prepared by solubilizing cells in RIPA buffer supplemented with Complete Mini protease inhibitor cocktail (Roche Diagnostics; Indianapolis, IN, USA), followed by a freeze-and-thaw cycle, and clarification with centrifugation. Protein concentrations were quantified using the Micro BCA assay (ThermoFisher). Equal amounts of protein (25 μg) were resolved using 10% SDS-PAGE and immunoblotted according to standard protocols. The membranes were blocked with 5% non-fat milk prepared in Tris-buffered saline containing 0.1% Tween-20 (TBST) for 1 hr, followed by incubation with primary antibodies prepared in TBST at 4 °C overnight. Primary antibodies used in this study include: CD36 (SMφ) (Santa Cruz Biotechnology), ATGL (30A4) (Cell Signaling; Danvers, MA, USA), PPARγ (C26H12) (Cell Signaling), and phospho-β-catenin (D2F1) (Cell Signaling). Secondary antibody was peroxidase-conjugated goat anti-mouse or rabbit IgG (1:10000, Jackson ImmunoResearch; West Grove, PA, USA) prepared in TBST. The membranes were incubated at room temperature for 1 hr. Protein bands were visualized using Immobilon Western chemiluminescent HRP substrate (EMD Millipore; Billerica, MA, USA). The blots were stripped and reprobed with rabbit anti-GAPDH (FL-335) (Santa Cruz Biotechnology) to ensure equal loading.

### 4.5. Cell Viability Assay

The relative viable cell number was evaluated using the colorimetric Cell Counting Kit-8 assay (CCK-8, Sigma-Aldrich), according to the manufacturer’s instructions. The absorbance at 450 nm was measured using a Victor IV microplate reader (PerkinElmer, Waltham, MA, USA).

### 4.6. PEDF Promoter Assay

The promoter region of the PEDF gene from positions −1642 to +107 was amplified by PCR using genomic DNA isolated from Hep3B cells, and cloned into the promoterless luciferase reporter vector pGL4.17 (Promega; Madison, WI, USA), using the *Xho*I/*Bgl*II restriction sites. The DNA inserts were verified by sequencing. For the reporter assay, AdSCs were seeded at 105 in 12-well plates. On the next day, cells were co-transfected using Lipofectamine 3000 transfection reagent (ThermoFisher) with 0.5 μg of the reporter construct and 0.5 μg pRL-SV40 (Promega), a plasmid that encodes Renilla Luciferase, as an internal control. After 6 hr, cell were starved in DMEM with 2% FBS overnight, followed by treatment with 1 μM Dex (in triplicates) for 48 hr before harvest. Firefly and Renilla luciferase activities were measured in cell suspension using the Dual-Glo Luciferase Reporter Assay System (Promega) on a VICTOR X4 multi-reader (PerkinElmer; Waltham, MA, USA), as directed by the manufacturer’s instructions. Relative promoter activities were calculated as the ratio of firefly/Renilla luciferase units. Data were presented as mean ± SD.

### 4.7. Statistical Analysis

For comparison between two groups, a two-tailed Student’s *t*-test was used. To compare between multiple measurements, differences were calculated by one-way analysis of variance (ANOVA) followed by Dunn’s post hoc test using GraphPad Prism (GraphPad Software; San Diego, CA, USA). The results are shown as mean ± standard deviation. A value of *p* < 0.05 was considered statistically significant.

## Supplementary Materials

Supplementary Materials can be found at http://www.mdpi.com/1422-0067/19/12/3992/s1.

## Abbreviations

PEDF	pigment epithelium-derived factor
ATGL	adipose triglyceride lipase
AdSCs	adipose-derived stem cells
CD36	cluster of differentiation 36
PPARγ	peroxisome proliferator-activated receptor gamma
C/EBP-α	CCAAT/enhancer-binding protein-alpha
SSO	sulfo-N-succinimidyl oleate
FASN	fatty acid synthase
SCD1	stearoyl-CoA desaturase 1
ACC1	acetyl-CoA carboxylase 1
NAFLD	non-alcoholic fatty liver disease
IBMX	3-isobutyl-1-methylxanthine
ON	osteonectin
