# Supplementary material for: Decreased PEDF Expression Promotes Adipogenic Differentiation through the Up-Regulation of CD36
Source: Int J Mol Sci. 2018 Dec 11;19(12):3992. doi: 10.3390/ijms19123992 (PMC6321369; doi:10.3390/ijms19123992)
Supplement: Supplementary file 1 [file ijms-19-03992-s001.pdf]

International Journal of *Molecular Sciences*

MDPI

Article

Decreased PEDF expression promotes adipogenic differentiation through up-regulation of CD36.

Kuang-Tzu Huang [1,2,*], Li-Wen Hsu [2], Kuang-Den Chen [1,2], Chao-Pin Kung [1,2], Shigeru Goto [3], and Chao-Long Chen [2]

[1] Institute for Translational Research in Biomedicine, Kaohsiung Chang Gung Memorial Hospital, Kaohsiung 83301, Taiwan; dennis8857@gmail.com (K.-D.C.); vina920715@gmail.com (C.-P.K.)
[2] Liver Transplantation Center, Department of Surgery, Kaohsiung Chang Gung Memorial Hospital, Kaohsiung 83301, Taiwan; hsuliwen1230@gmail.com (L.-W.H.); clchen@cgmh.org.tw (C.-L.C.)
[3] Fukuoka Institution of Occupational Health, Fukuoka 815-0081, Japan; pochigoto0224@gmail.com (S.G.)
* Correspondence: huangkt@cgmh.org.tw; Tel.: +886-7-731-7123 ext. 8193

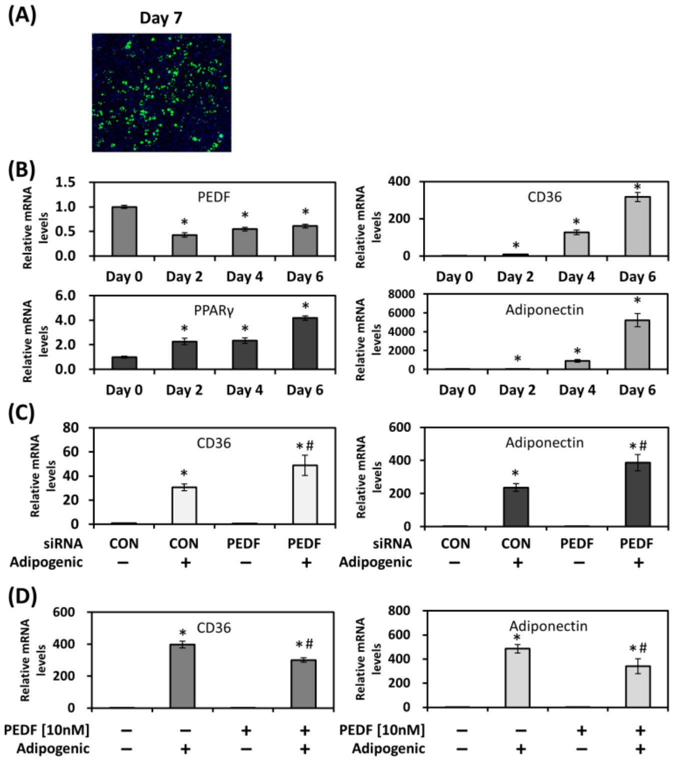

Figure S1. Decreased PEDF is associated with CD36 up-regulation during adipogenic differentiation of mouse 3T3-L1 cells. (A) Differentiated mouse 3T3-L1 cells were fixed, stained with BODIPY 493/503 and counterstained with DAPI. A representative fluorescence micrograph is shown. (B) Gene expression of PEDF, CD36, PPARγ and adiponectin in differentiating 3T3-L1 cells was measured using quantitative RT-PCR. (C) Gene expression was evaluated in differentiated 3T3-L1 cells transfected with control or PEDF siRNA. (D) Gene expression was determined in differentiated 3T3-L1 cells in the absence or presence of recombinant PEDF. *, statistically significant compared with the control transfected, vehicle treated group at $p<0.05$; #, statistically significant compared with the PEDF siRNA/PEDF protein treated, vehicle treated group at $p<0.05$.

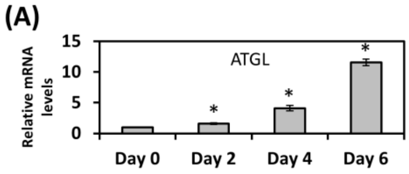

Figure S2. ATGL is up-regulated during adipogenic differentiation of 3T3-L1 cells. (A) ATGL expression was measured in differentiating 3T3-L1 cells using quantitative RT-PCR. *, statistically significant compared with the control group at $p<0.05$.

Table S1. List of siRNAs used in this study.

Gene name	Manufacturer	Species	Cat No.	Assay ID
PEDF	ThermoFisher	Mouse	4390771	s73477
CD36	ThermoFisher	Mouse	4390771	s63620
PEDF	ThermoFisher	Rat	4390771	s142621
Neg Ctrl [1]	ThermoFisher	-	4390843	-

[1] Neg Ctrl: negative control

Table S2. List of primers used in this study.

Gene name	Species	Forward primer	Reverse primer
PEDF	Rat	CCAACTCTTTGCAGGACATG	TCACAGGTTTGCCGGTAATC
PPARγ	Rat	CTGTCGGTTTCAGAAGTGCCTT	AGCTGGTCGATATCACTGGAGA
C/EBP-α	Rat	TCACTTGCAGTTCCAGATCG	TTGACCAAGGAGCTCTCAGG
adiponectin	Rat	GATACCGGGCCGTGATGG	CCCTTCCGCTCCTGTCATTC
CD36	Rat	GCCTCCTTTCCACCTTTTGT	GATTCAAACACAGCATAGATGGAC
ATGL	Rat	TGTGGCCTCATTCCTCCTAC	TGAGAATGGGGACACTGTGA
ON	Rat	CTGCCACTTCTTTGCGACCA	CTCCAGGCGCTTCTCGTTCTC
GAPDH	Rat	TTCTAGAGACAGCCGCATCT	TGGTAACCAGGCGTCCGATA
PEDF	Mouse	ACGATACGGCTTGGACTCTG	GTCAAGTTCTGGGTCACGGT
PPARγ	Mouse	AGAGGGCCAAGGATTCATGACCAGG	TTCAGCTTGAGCTGCAGTTCCAGGG
adiponectin	Mouse	CGGCAGCACTGGCAAGTTCTACTGC	TTGTGGTCCCCATCCCCATACACCT
CD36	Mouse	TGGCCAAGCTATTGCGACAT	TTCAGATCCGAACACAGCGT
ATGL	Mouse	GCCAACGCCACTCACATCTA	AATGTTGGCACCTGCTTCAC
FASN	Mouse	CCAAGCAGGCACACACAATG	GTTCGTTCCTCGGAGTGAGG
SCD1	Mouse	CCAAGCTGGAGTACGTCTGGA	AGAGCGCTGGTCATGTAGTAGA
ACC1	Mouse	GGAGATGTACGCTGACCGAG	TACCCGACGCATGGTTTTCA
β-actin	Mouse	TGTCCACCTTCCAGCAGATGT	AGCTCAGTAACAGTCCGCCTAGA
PEDF Pm [1]	Human	ATCACGCGTAGAGCAAGGTTCCATCTCAAA	ATCAGATCTACACCCAGCCTAGTCCCTCTA

[1] PEDF Pm: PEDF promoter
